# Progression to AIDS in South Africa Is Associated with both Reverting and Compensatory Viral Mutations

**DOI:** 10.1371/journal.pone.0019018

**Published:** 2011-04-22

**Authors:** Kuan-Hsiang Gary Huang, Dominique Goedhals, Jonathan M. Carlson, Mark A. Brockman, Swati Mishra, Zabrina L. Brumme, Stephen Hickling, Christopher S. W. Tang, Toshiyuki Miura, Chris Seebregts, David Heckerman, Thumbi Ndung'u, Bruce Walker, Paul Klenerman, Dewald Steyn, Philip Goulder, Rodney Phillips, Bloemfontein-Oxford Collaborative Group, Cloete van Vuuren, John Frater

**Affiliations:** 1 Nuffield Department of Clinical Medicine, Oxford University, Oxford, United Kingdom; 2 University of Free State, Bloemfontein, South Africa; 3 National Health Laboratory Services (NHLS), Johannesburg, South Africa; 4 eScience Group, Microsoft Research, Los Angeles, California, United States of America; 5 Simon Fraser University, Burnaby, British Columbia, Canada; 6 British Columbia Centre for Excellence in HIV/AIDS, Vancouver, British Columbia, Canada; 7 Ragon Institute of Massachusetts General Hospital, Harvard University and Massachusetts Institute of Technology (MIT), Boston, Massachusetts, United States of America; 8 Institute of Medical Science, University of Tokyo, Tokyo, Japan; 9 Biomedical Informatics Research Division, Medical Research Council, Cape Town, South Africa; 10 HIV Pathogenesis Programme, The Doris Duke Medical Research Institute, University of KwaZulu-Natal, Durban, South Africa; 11 Infectious Disease, Massachusetts General Hospital, Boston, Massachusetts, United States of America; 12 Division of AIDS, Harvard University, Boston, Massachusetts, United States of America; 13 Department of Paediatrics, University of Oxford, Oxford, United Kingdom; 14 The James Martin 21st Century School, Oxford, United Kingdom; 15 Oxford NIHR Biomedical Research Centre, Oxford, United Kingdom; University of South Carolina School of Medicine, United States of America

## Abstract

We lack the understanding of why HIV-infected individuals in South Africa progress to AIDS. We hypothesised that in end-stage disease there is a shifting dynamic between T cell imposed immunity and viral immune escape, which, through both compensatory and reverting viral mutations, results in increased viral fitness, elevated plasma viral loads and disease progression. We explored how T cell responses, viral adaptation and viral fitness inter-relate in South African cohorts recruited from Bloemfontein, the Free State (n = 278) and Durban, KwaZulu-Natal (n = 775). Immune responses were measured by γ-interferon ELISPOT assays. HLA-associated viral polymorphisms were determined using phylogenetically corrected techniques, and viral replication capacity (VRC) was measured by comparing the growth rate of gag-protease recombinant viruses against recombinant NL4-3 viruses. We report that in advanced disease (CD4 counts <100 cells/µl), T cell responses narrow, with a relative decline in Gag-directed responses (p<0.0001). This is associated with preserved selection pressure at specific viral amino acids (e.g., the T242N polymorphism within the HLA-B*57/5801 restricted TW10 epitope), but with reversion at other sites (e.g., the T186S polymorphism within the HLA-B*8101 restricted TL9 epitope), most notably in Gag and suggestive of “immune relaxation”. The median VRC from patients with CD4 counts <100 cells/µl was higher than from patients with CD4 counts ≥500 cells/µl (91.15% versus 85.19%, p = 0.0004), potentially explaining the rise in viral load associated with disease progression. Mutations at HIV Gag T186S and T242N reduced VRC, however, in advanced disease only the T242N mutants demonstrated increasing VRC, and were associated with compensatory mutations (p = 0.013). These data provide novel insights into the mechanisms of HIV disease progression in South Africa. Restoration of fitness correlates with loss of viral control in late disease, with evidence for both preserved and relaxed selection pressure across the HIV genome. Interventions that maintain viral fitness costs could potentially slow progression.

## Introduction

With few exceptions, untreated individuals infected with Human Immunodeficiency Virus Type 1 (HIV-1) develop Acquired Immunodeficiency Syndrome (AIDS), associated with opportunistic infections, malignancies and, eventually, death. Some patients progress to AIDS quickly, whilst others maintain undetectable plasma viral loads without therapy and do not become unwell for many years. Deciphering the correlates of this heterogeneous protection is important, as there are implications for the design of vaccines and other interventions.

The pace of HIV disease progression is multifactorial - a mixture of host and pathogen genetics combined with factors such as the immune response and viral adaptation. In genome-wide association studies a limited number of SNPs and alleles correlate with lower viral loads [1] [2], and HLA Class I and the human MHC associate reproducibly [3]. The role of the cell-mediated immune system in HIV-associated disease has received much scrutiny, especially the effect of different HLA Class I alleles. Well-documented examples include the protection conferred by HLA B*57 and B*27 [4] [5] in Caucasian individuals and HLA B*5801 and B*8101 in patients from South Africa [6].

What determines this differential HLA Class I effect is unclear. ‘Beneficial’ HLA Class I alleles may be associated with T cell clones with broader cross-reactivity to viral variants due to reduced thymic selection [7], and thus broader and more pervasive selection pressure. However, HIV is adept at adapting to selection pressures invoked by both antiretroviral drugs (ARVs) and the immune system in the forms of drug resistance [8] [9] and immune escape mutations [10] [11], respectively. The latter are widespread across the HIV-1 genome [12] [13] [14], and may influence outcome in individual patients [15] and across different populations [16].

Escape from an effective immune response is determined by the strength of the imposed selection pressure and may explain why the prevalence of HLA-associated polymorphisms is greater for HLA Class I alleles associated with protection [17]. Although the adapted virus maintains a fitness advantage in the presence of the selection pressure conferred by cytotoxic T cells (CTL), there may be a significant drop in replicative capacity compared to a wild-type virus in a selection-free environment [18] [19]. We, and others, have previously hypothesised that immune escape may therefore result in the maintenance of relatively lower viral loads and clinical advantage [17] [20], [21]. This is supported by high reversion rates of escape mutations selected by ‘beneficial’ HLA Class I alleles following transmission to HLA-mismatched recipients [22] [23].

These interactions between HLA Class I imposed selection, viral escape and viral fitness are together likely to influence clinical progression, however the mechanisms that lead to progression to late disease and AIDS are poorly defined. We hypothesised that the nature of these interactions can be understood better by investigating patients with late-stage HIV infection to determine if, or how, CD8+ve T cells are maintaining selection pressure, and whether the virus shows different patterns of adaptation and fitness costs compared to patients with earlier infection. There are limited reports that, despite the loss of CD4+ve cells, CD8+ve T cells may still be functional in AIDS, although with varied avidity, less polyfunctionality and less differentiation [24], and targeting Env rather than Gag [25]. We proposed that if CD8 T cell pressure is ‘relaxed’ due to HIV-induced immunodeficiency this might facilitate reversion of costly escape mutations, leading to a restoration of viral fitness and the subsequent rise in viraemia seen in AIDS. Reversion of costly drug resistance mutations has been associated with a rise in viral load and clinical progression [26], and therefore a precedent exists to potentially explain the rise in viral load associated with the onset of AIDS. Alternatively, CTL pressure may be maintained, but the virus might develop secondary compensatory mutations, which restore the replicative cost of the initial escape mutation. Compensatory mutations have been reported in chronic infection [27], [28], but whether they explain the AIDS-associated rise in viraemia is not known.

In this study we question whether progression to AIDS is associated with an increase in viral fitness, and whether this is related to changes in T cell imposed selection pressure. We start by comparing T cell ELISPOT responses in patients across different CD4 count strata, and then examine how the variation in these responses correlates with different patterns of selection across the HIV genome in patients with very low CD4 cell counts compared with less progressed infections. Finally, we measure the viral fitness of these variants to correlate the replicative cost of adaptation with outcome. We investigated 1053 untreated HIV-1 infected South African individuals from Bloemfontein and Durban, and found that viral fitness was greater in patients with advanced disease with examples of both viral compensatory mutations and of reduction in escape mutations, the latter possibly due to ‘immune relaxation’. Despite the complex interplay between host and pathogen, these data support a key role for the restoration of viral fitness in association with AIDS progression and help to explain the rise in viraemia in terminal stages.

## Methods

### Ethics Statement

Full ethical approval was gained for the study of both cohorts. Written informed consent was provided by all study participants. The study ethics for the Bloemfontein cohort was approved by the University of the Free State (ETOVS 10/04 and ETOVS 206/05) and for the Durban cohort by the University of KwaZulu-Natal Review Board.

### Study subjects

Participants from two South African cohorts were studied (total n = 1053) - the ‘Bloemfontein’ cohort from the Free State (n = 278) and the ‘Durban’ cohort from KwaZulu-Natal (n = 775). Both are established cohorts, described elsewhere ([29] [6]). In summary, each of the cohorts was comprised of antiretroviral naïve and chronically HIV-1 subtype C-infected adults from neighbouring provinces in the central east region of South Africa. Of these, 916 patients with data on CD4 cell count, HLA class I type and either HIV *gag*, *pol* or *nef* sequences were included in the analysis of HLA-linked polymorphisms, and comprised the ‘total’ cohort. Subsequent analyses involved stratification of the patients according to HLA type, plasma viral load and CD4 T cell count. In the CD4 T cell count stratification, patients were assigned to either “High CD4” (CD4 T cell counts >500 cells/µl, n = 299), or “Low CD4” (CD4 T cell counts <100 cells/µl, n = 196).

### HLA typing

Participants' HLA Class I type was determined to the oligo-allelic level using Dynal RELITM Reverse Sequence-Specific Oligonucleotide kits for the HLA-A, -B and –C loci (Dynal Biotech). To obtain four-digit typing, Dynal Biotech Sequence-Specific priming kits were used, in conjunction with the Sequence-Specific Oligonucleotide type.

### T Cell ELISPOT assays

Peptides, 18 amino acids in length (n = 410), overlapping by 10 amino acids, and spanning the entire expressed HIV genome, were synthesized based on the consensus of available C-clade sequences in 2001. These peptides were used in a ‘mega-matrix’ of 11–12 peptides per pool to test patient samples for HIV-specific T cell responses by interferon-gamma ELISPOT assay, as previously described [30]. Confirmation of recognised individual 18-mer peptides within a peptide pool was carried out in separate ELISPOT assays.

### Sequencing of HIV *gag*, *pol* and *nef*

HIV *gag*, *pol* and *nef* were sequenced using previously described methods and primers [17]. In brief, viral RNA was extracted from plasma, reverse transcribed to cDNA and amplified using a nested polymerase chain reaction (PCR) protocol. Population sequencing of the HIV pol gene was carried out using ABI Big Dye terminator sequencing kits (Applied Biosystems), according to manufacturer's instructions. Sequences were aligned manually using X11 and Se-Al software.

### Identification of HLA class I linked polymorphisms

Amino acid polymorphisms that associated with host HLA class I alleles, were identified using previously described methods utilising ‘phylogenetic dependency networks’ [31]. Briefly, the analysis combines phylogenetic correction with a statistical model of evolution to evaluate associations between host HLA class I alleles and viral amino acid site-specific polymorphisms. The analysis adjusts for confounding factors including founder effects, linkage disequilibrium of host HLA types, co-variation in HIV codons and corrections for multiple statistical tests. The significance of an association is expressed as a ‘q value’, which estimates the false discovery rate for each p-value. In this study an association is considered significant for q<0.2. ‘Escape’ describes increased polymorphisms observed at a particular amino acid site in the presence of a specific HLA class I allele. ‘Reversion’ describes decreased polymorphisms observed at a particular amino acid site in the absence of a specific HLA class I allele. The strength of associations between HLA class I alleles and viral polymorphisms in the “total” cohort, “high CD4” group or “low CD4” group was derived using a logistic regression model that corrected for phylogeny, and is reported as a log_2_-adjusted odds ratio.

Identified polymorphisms were mapped to known cytotoxic T cell epitopes. Epitope maps were defined from experimental data from the Durban cohort [6], and from those listed in the A-list of Los Alamos Database (http://www.hiv.lanl.gov/content/immunology/pdf/2008/optimal_ctl_article.pdf). The epitope flanking region was also included in the analysis, defined according to the five amino acids neighbouring to the defined epitope in both C and N terminal directions.

### Generation of chimeric NL4-3 viruses

Chimeric viruses were constructed by recombining a *gag-protease*-deleted pNL4-3 HIV plasmid (pNL4-3Δ*gag-protease*) with autologous *gag-protease* amplified from virus extracted from patient plasma, as described elsewhere [19], [32], [33], [34].

In brief, to amplify the autologous HIV *gag-protease* viral RNA was extracted from plasma, reverse transcribed and amplified using specific primers in the first round of nested PCR using the Superscript III One-step RT-PCR kit with high fidelity Platinum *Taq* polymerase (Invitrogen) (forward primer: 5′ AAATCTCTAGCAGTGGCGCCCGAACAG 3′, HXB2 nucleotides 623 to 649; reverse primer: 5′ TTTAACCCTGCTGGGTGTGGTATYCCT 3′, HXB2 nucleotides 2851 to 2825). The second round of the nested PCR was conducted using a 100-mer primer pair (forward primer: 5′ GACTCGGCTTGCTGAAGCGCGCACGGCAAGAGGCGAGGGGCGGCGACTGGTGAGTACGCCAAAAATTTTGACTAGCGGAGGCTAGAAGGAGAGAGATGGG 3′, HXB2 nucleotides 695 to 794; reverse primer: 5′ GGCCCAATTTTTGAAATTTTTCCTTCCTTTTCCATTTCTGTACAAATTTCTACTAATGCTTTTATTTTTTCTTCTGTCAATGGCCATTGTTTAACTTTTG 3′, HXB2 nucleotides 2704 to 2605) that completely matched the pNL4-3 sequences using Platinum *Taq* polymerase (Invitrogen).

The pNL4-3 HIV plasmid had been mutated to contain unique restriction enzyme (*BstEII*) sites at the 5′ end of the *gag* gene and 45 nucleotides downstream from the 3′ end of the *protease* gene, as described elsewhere [19]. The *BstEII* enzyme digest results in deletion of HIV *gag-protease* and self ligation of the remaining plasmid (pNL4-3Δ*gag-protease*). For transfection, the pNL4-3Δ*gag-pro*tease was linearised by *BstEII* digestion for two hours at 60°C (10 U/µl of enzyme per 1000 µg/mL of plasmid). For each sample, 10 µg of the linearised pNL4-3Δ*gag-pro*tease was co-transfected with 5 µg of patient-derived *gag-protease* PCR product in 3.9×10^6^ tat-driven GFP reporter T cells (GXR cells of CEM origin). After transfection by electroporation (250 V, 950 µF, for 30–40 msec), 10 µl polybrene (4 µg/µl) and 1×10^6^ GXR cells were added and incubated at 37°C. Positive control samples were generated using co-transfection of pNL4-3Δ*gag-pro*tease with *gag-protease* amplified from pNL4-3, pHXB2 (subtype B), and pMJ4 (subtype C). After five days, GFP expression was measured every 1∼2 days using a FACSCalibur flow cytometer (FACSCalibur; BD Biosciences, San Jose, CA). Supernatant was harvested when >15% of cells were positive for GFP, and stored at −80°C.

### Measurement of viral replication capacity (VRC)

Viral titration was performed in 1×10^6^ GXR cells to determine the multiplicity of infection (MOI). The VRC was assayed by adding recombinant virus supernatant to 1×10^6^ GXR cells at an MOI of 0.003. The VRC was measured, at least in duplicate, using gating strategies as previously described [19]. The growth of virus was calculated from the slope derived from the natural log of the percentage of GFP positive cells between days 2 and day 7, as appropriate for an exponential growth curve. The final VRC of each variant is expressed as percentage growth rate of the variants compared to that of recombinant NL4-3 strains. In addition, for each VRC assay, viral supernatants were collected on day 7 for viral RNA extraction and sequencing to confirm the population sequences VRC measured.

### Statistics

Student t-tests (two tailed) and Mann-Whitney tests (two tailed) were used to compare various clinical parameters and VRC measurements. The statistical analyses were carried out using Prism4 for Macintosh (GraphPad Software, version 4.0c). Associations between HLA Class I alleles and viral polymorphisms were determined using phylogenetically-corrected algorithms, as described above.

## Results

### Structure of cohorts

Patients were recruited from the Bloemfontein and Durban cohorts [29] [6]. The Bloemfontein cohort comprises 884 drug-naïve patients at all stages of HIV infection recruited through the South African government ARV program. Plasma samples were studied from 278 out of the 884 participants: 96 out of 110 patients in the high (CD4 count>500 cells/µl), 18 out of 308 in an intermediate (CD4 count = 201–400 cells/µl), and 164 of 183 in the low (CD4 count<100 cells/µl) strata. The samples were used for the HLA-association study and to make recombinant viral strains for the analysis of fitness. To increase the power of the HLA-association analysis we incorporated HIV viral sequences from the neighbouring Durban cohort, which comprised 775 adults, naive to antiretroviral therapy. Of these, 219 were asymptomatic women identified through antenatal clinics. The remaining 556 subjects were recruited from out-patient clinics. For all 775 patients host HLA class I alleles were genotyped. ELISPOT assays were carried out using overlapping peptides designed from the subtype C majority consensus. For 656 of these patients, sequences of either viral *gag*, *pol* and *nef* genes were available.

### Breadth of ELISPOT responses narrows in advanced disease

**Figure 1:**
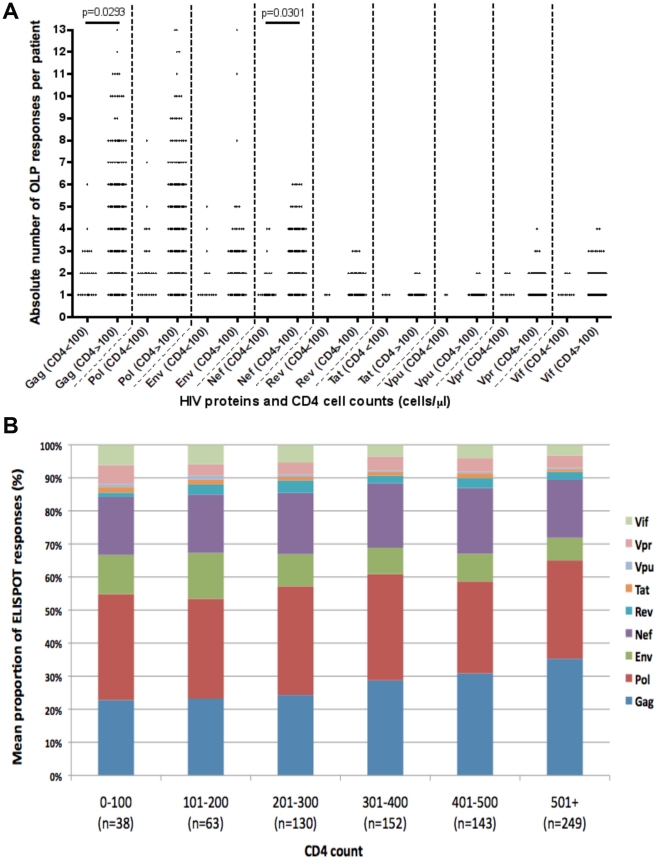
Gamma interferon ELISPOT responses in patients with CD4 counts above and below 100 cells/µl. Recognition of overlapping peptides covering the full HIV genome is shown in patients with CD4 cell counts above and below 100 cells/µl. ELISPOT responses are displayed as (a.) the absolute breadth of responses, reflecting the mean number of overlapping peptides recognised by each patient for each HIV protein and, (b.) the relative breadth, reflecting the proportion of ELISPOT responses targeted against different HIV proteins in patients within different CD4 count strata.

In order to measure the T cell-imposed selection pressure acting on HIV at different stages of the infection, ELISPOT assays using overlapping peptides (OLPs) covering the entire HIV genome were analysed from 775 patients. Results were stratified according to CD4 cell count and are presented according to the number of OLPs seen per patient for each gene (Figure 1a), and the percentage contribution of each protein to all responses in each strata (Figure 1b). The figure shows that over the course of infection, there is a general narrowing of the absolute breadth of responses (Figure 1a). However, the relative contribution of Gag to the total responses decreased (p<0.0001, r^2^ = 0.055) in contrast to the proportion of responses to other proteins such as Env (p<0.0001, r^2^ = 0.024) and Vif (p = 0.003, r^2^ = 0.011), which was greater in patients with lower CD4 cell counts. We hypothesized that the reduction in breadth of CD8+ve T cell responses might result in, and therefore correlate with, a decline in selection pressure, particularly in Gag in light of the relative shift away from Gag targeting. As a result, this ‘immune relaxation’ might allow the reversion of costly viral escape mutations back to a fitter wild type state. Accordingly, we next analysed HLA Class I-associated polymorphisms in the HIV-1 *gag*, *pol* and *nef* genes to identify any difference in the strength of selection pressure in patients with high and low CD4 cell counts.

### Identification of HLA class I linked polymorphisms in HIV-1 *gag, pol and nef*

To identify evidence of differential HLA Class I imposed selection pressure in patients with high and low CD4 cell counts, we carried out a study to identify HLA Class I associations with particular amino acid polymorphisms in this cohort. We accounted for potential confounding effects such as phylogeny, founder effect and multiple comparisons by using phylogenetic dependency networks [31] - an approach which reports associations between specific viral polymorphisms and HLA Class I alleles according to p value, q value (a measure of false positive error) and a phylogenetically-adjusted odds ratio.

Initially, we analysed 916 HIV-1 *gag*, *pol* and *nef* sequences from the combined Durban and Bloemfontein cohorts and then we carried out sub-group analyses on patients with either ‘high’ (>500 cells/µl, n = 299) or ‘low’ (<100 cells/µl, n = 196) CD4 cell counts. We then selected those HIV amino acid polymorphisms significantly associated with an HLA Class I allele in either, or both of, the ‘high’ or ‘low’ CD4 count group of patients for further analysis. The results for *gag*, *pol* and *nef* are presented in Tables 1, 2 and 3, respectively. The tables detail each viral polymorphism and its associated HLA Class I allele, whether the association lies in, or within, the flanking region of a known restricted epitope and whether this association is expected to revert in HLA-mismatched hosts [35]. The q values for the statistically significant associations for the whole cohort, the ‘high’ CD4 count subgroup (>500 cells/µl) and ‘low’ CD4 count subgroup (<100 cells/µl) are shown, followed by the log_2_-adjusted odds ratios for the ‘high’ and ‘low’ groups. In the final column, the p value is reported, when there is a significant difference between the two odds ratios.

**Table 1:** HLA-related associated polymorphisms in the HIV Gag protein in different CD4 count strata.

						q-value of HLA-related association in each strata			Phylogentically corrected Log2 Odds Ratio (pLOR) towards “escape”		
HIV-1 protein	HLA Class I	Population majority consensus amino acid at associated site	HXB2 site and direction of HLA associated polymorphism	Is the amino acid part of known epitope (E), or within flanking (F) region (within 5 amino acid)	Is there evidence for reversion in HLA mismatched host (R)?	Entire population	High CD4 cell count strata (>500 cells/µl)	Low CD4 cell count strata (<100 cells/µl)	High CD4 cell count strata (>500 cells/µl)	Low CD4 cell count strata (<100 cells/µl)	p-value (likelihood ratio test between pLOR of high and low CD4 strata)
Gag p17	A29	I	I7V				0.189				
Gag p17	A74	K	K12N/Q		R	0.000	0.000		18.83	- (infinity)	0.0070
Gag p17	B5801	K	K12Q		R		0.147				
Gag p17	A3303	K	K15S			0.026	0.062				
Gag p17	A6801	K	T15	F		0.131	0.086				
Gag p17	B45	H	H28R					0.058	- (infinity)	15.05	0.0005
Gag p17	C17	H	H28Q			0.000	0.183	0.017			
Gag p17	B4201	M	M30	E		0.004	0.122		14.14	9.44	0.0443
Gag p17	B0801	A	A45S		R			0.128	- (infinity)	5.29	0.0051
Gag p17	A6802	E	E55D					0.015	−6.35	18.13	0.0009
Gag p17	A3402	K	Q62				0.149		(infinity)	−10.24	0.0130
Gag p17	A3001	K	K62S				0.183		10.89	- (infinity)	0.0151
Gag p17	A30	Q	Q65H		R		0.086		9.27	−11.37	0.0086
Gag p17	B8101	Q	K69				0.163		(infinity)	−0.21	0.0027
Gag p17	A26	K	K76R				0.149				
Gag p17	B15	Y	79Y		R		0.188		14.22	−1.66	0.0193
Gag p17	C04	K	K91N		R			0.016	15.78	4.21	0.0004
Gag p17	B08	Q	Q112K				0.159				
Gag p17	B1402	K	K114				0.069		7.73	- (infinity)	0.0498
Gag p17	B1801	T	T115				0.146				
Gag p17	A2902	Y	Y132F				0.188				
Gag p24	B57	A	A146P	E		0.000	0.000	0.048			
Gag p24	C17	A	P146		R		0.102				
Gag p24	B1510	A	A146	E		0.008	0.153				
Gag p24	C0304	I	147I	E			0.102		(infinity)	1.41	0.0209
Gag p24	B57	I	I147L/M	E	R	0.000	0.000				
Gag p24	C17	Q	Q182					0.017	0.79	4.18	0.0019
Gag p24	B8101	Q	Q182S	E		0.000	0.000	0.017	13.97	16.79	0.0349
Gag p24	B8101	T	T186S	E	R	0.000		0.000	18.38	7.25	0.0397
Gag p24	C0210	V	V218		R			0.056			
Gag p24	B5801	T	T242N	E		0.000	0.000	0.000			
Gag p24	B57	T	T242N	E	R	0.000	0.000	0.000			
Gag p24	A74	I	I247		R		0.087				
Gag p24	C04	S	N252				0.189				
Gag p24	B35	D	D260E	E		0.000	0.000				
Gag p24	B1401	K	K302R	E	R	0.000	0.015				
Gag p24	B44	D	D312E	E	R	0.000	0.183				
Gag p24	B0702	D	D319E				0.086				
Gag p24	C08	T	T332A			0.131		0.015			
Gag p24	B0702	G	S357G	E		0.000	0.001	0.000			

The table shows the results of the association analysis between HLA Class I alleles and HIV viral polymorphisms in HIV Gag. The columns of the table detail the HIV protein, the associated HLA Class I allele and viral polymorphism, whether the association lies in, or within, the flanking region of a known restricted epitope and whether this association is expected to revert in HLA-mismatched hosts. The q values for the statistically significant associations for the whole cohort, the ‘high’ CD4 count subgroup (>500 cells/µl) and ‘low’ CD4 count subgroup (<100 cells/µl) are shown, followed by the log_2_-adjusted odds ratios for the ‘high’ and ‘low’ groups. In the final column, the p value is reported, where there is a significant difference between the two odds ratios.

**Table 2:** HLA-related associated polymorphisms in the HIV Pol protein in different CD4 count strata.

						q-value of HLA-related association in each strata			Phylogentically corrected Log2 Odds Ratio (pLOR) towards “escape”		
HIV-1 protein	HLA Class I	Population majority consensus amino acid at associated site	HXB2 site and direction of HLA associated polymorphism	Is the amino acid part of known epitope (E), or within flanking (F) region (within 5 amino acid)	Is there evidence for reversion in HLA mismatched host (R)?	Entire population	High CD4 cell count strata (>500 cells/µl)	Low CD4 cell count strata (<100 cells/µl)	High CD4 cell count strata (>500 cells/µl)	Low CD4 cell count strata (<100 cells/µl)	p-value (likelihood ratio test between pLOR of high and low CD4 strata)
Protease	C0401	L	L10		R		0.178				
Protease	C18	S	S12T					0.010	2.13	16.45	0.0005
Protease	B5802	S	T12S			0.000		0.007			
Protease	C06	S	12S					0.007			
Protease	B1801	I	I19T					0.162			
Protease	B1510	E	D35E				0.155		−5.82	20.55	0.0015
Protease	B44	E	E35D	E		0.000		0.000			
Protease	A6601	N	N37			0.167		0.121			
Protease	C0602	L	V63T	E		0.192		0.190	13.30	(infinity)	0.0044
Protease	C16	L	P63L		R			0.013			
Protease	C18	L	P63S		R	0.000		0.064			
Protease	C08	L	63L					0.100			
Protease	A43	I	I64L		R	0.167		0.097	- (infinity)	20.66	0.0477
Protease	C06	V	I77V			0.004		0.013	3.60	6.41	0.0129
Protease	A66	V	V77I					0.196			
RT	B81	P	P103S/T	E		0.000	0.155	0.000	10.43	22.85	0.0092
RT	C18	E	E105K			0.001	0.178		10.97	−1.39	0.0207
RT	B81	E	E105	E		0.000	0.178				
RT	B18	K	K119R		R			0.073			
RT	B15	E	E152D					0.035			
RT	B4101	E	E152D			0.141		0.080			
RT	A34	V	V159I					0.121	0.54	17.36	0.0155
RT	B35	E	E221K	E		0.003	0.178				
RT	C02	G	S222			0.002		0.015			
RT	B8101	I	I234R					0.131	−5.66	22.55	0.0379
RT	B4202	I	I234V					0.036			
RT	B0702	S	S261A/C	E		0.000		0.041	15.26	16.55	0.0348
RT	C0702	S	S261					0.029			
RT	B07	T	T264I	E		0.000		0.000			
RT	A03	K	K265R	E	R	0.005		0.121			
RT	C0702	E	E268					0.010			
RT	B1503	K	K273R	E		0.002		0.003	−14.09	18.11	0.0302
RT	A03	K	K273R	F		0.000		0.035	15.22	18.75	0.0444
RT	A80	E	E276D			0.171		0.092			
RT	B4403	E	E306K/N/R	E	R	0.000		0.001			
RT	B1801	E	E306			0.167		0.131			
RT	C06	E	D306					0.143			
RT	C04	K	R310K		R			0.072	16.98	17.95	0.0001
RT	A74	K	K310		R		0.178		16.39	−10.22	0.0059
RT	A80	D	D349					0.195			
RT	A30	I	I356		R			0.121			
RT	A33	P	P371S					0.100	−12.13	5.66	0.0196
RT	B4201	P	P371A	E				0.000	12.06	15.09	0.0040
RT	C1701	P	P371A/S			0.000	0.054				
RT	B42	I	I373V	E	R	0.000		0.037			
RT	A3001	K	K374	F				0.064			
RT	A03	R	K376R	E		0.000		0.006			
RT	A3001	K	K380R			0.000		0.005			
RT	B57	A	A385V				0.178				
RT	C0210	A	A385T				0.198				
RT	B15	A	A385V		R			0.099			
RT	B45	E	E396K					0.153			
RT	C04	D	E423D		R	0.057		0.143			
RT	B4403	I	I425V					0.003	2.39	20.64	0.0259
RT	C03	M	R456				0.178				
RT	B42	R	457R					0.110	−20.83	(infinity)	0.0039
RT	C06	R	R457K		R	0.000		0.019			
RT	A6601	R	R457K		R			0.097			
RT	B5802	T	458T		R			0.103			
RT	C03	A	A470		R			0.150			
RT	C0302	I	I474V		R	0.165		0.000	- (infinity)	(infinity)	0.0015
RT	B15	L	L476I		R			0.128			
RT	B5801	S	S478C/A	E	R	0.004		0.001	- (infinity)	16.17	0.0001
RT	A32	E	E498	E				0.080			
RT	A3201	T	T499I	E		0.001		0.010			
RT	A29	T	499T					0.081			
RT	B13	D	D503N			0.007	0.051				
RT	B39	A	A534V					0.033	- (infinity)	(infinity)	0.0018
RT	B5802	A	I534					0.196			
RT	A6802	A	A536V	E	R	0.007		0.053	15.20	15.75	0.0177
RT	A6601	A	A536V	F		0.001	0.178	0.038			
RT	A2301	N	N546H		R	0.073	0.165		12.30	−5.48	0.0030
RT	B4403	E	E548D					0.025			
RT	A3303	I	551I				0.198		(infinity)	- (infinity)	0.0000
RT	B5802	I	I551A		R			0.072			
RT	B4202	V	V566I					0.143			
RT	B08	V	V566I					0.145			
RT	B08	S	S567T			0.000		0.003			
RT	B08	L	L568					0.190			
RT	A3402	T	T569A		R	0.082	0.155				
RT	B18	Q	Q582H	E		0.001		0.073			
RT	B1503	S	590S	F	R			0.150	−12.70	5.01	0.0196
RT	B44	S	S590L/P	E	R	0.000		0.000	0.74	3.95	0.0408
RT	B1401	S	S590L	F			0.178				
RT	B4403	E	E591	E		0.018		0.011			
RT	B3910	I	I594V		R	0.000		0.005			
RT	B4202	K	K611N		R	0.071		0.120			
RT	B5802	L	L616I					0.009	−11.95	17.59	0.0084
RT	C0701	K	K626N					0.188	−5.73	17.64	0.0045
Integrase	B4403	E	E669A	E	R	0.000		0.011			
Integrase	B4403	E	E670D	E	R	0.000		0.000			

The table shows the results of the association analysis between HLA Class I alleles and HIV viral polymorphisms in HIV Pol. The columns of the table detail the HIV protein, the associated HLA Class I allele and viral polymorphism, whether the association lies in, or within, the flanking region of a known restricted epitope and whether this association is expected to revert in HLA-mismatched hosts. The q values for the statistically significant associations for the whole cohort, the ‘high’ CD4 count subgroup (>500 cells/µl) and ‘low’ CD4 count subgroup (<100 cells/µl) are shown, followed by the log_2_-adjusted odds ratios for the ‘high’ and ‘low’ groups. In the final column, the p value is reported, where there is a significant difference between the two odds ratios.

**Table 3:** HLA-related associated polymorphisms in the HIV Nef protein in different CD4 count strata.

						q-value of HLA-related association in each strata			Phylogentically corrected Log2 Odds Ratio (pLOR) towards “escape”		
HIV-1 protein	HLA Class I	Population majority consensus amino acid at associated site	HXB2 site and direction of HLA associated polymorphism	Is the amino acid part of known epitope (E), or within flanking (F) region (within 5 amino acid)	Is there evidence for reversion in HLA mismatched host (R)?	Entire population	High CD4 cell count strata (>500 cells/µl)	Low CD4 cell count strata (<100 cells/µl)	High CD4 cell count strata (>500 cells/µl)	Low CD4 cell count strata (<100 cells/µl)	p-value (likelihood ratio test between pLOR of high and low CD4 strata)
Nef	B07	I	I10R	F				0.178			
Nef	A01	E	E24A					0.172	- (infinity)	15.19	0.0024
Nef	B42	A	A26P		R	0.013		0.053			
Nef	A6802	A	A53E					0.134			
Nef	A0201	C	C55	F				0.033			
Nef	B45	E	E65D	E				0.150	- (infinity)	20.98	0.0498
Nef	B45	E	E66D	E		0.000		0.178			
Nef	C0702	R	R71K		R	0.000	0.110	0.000			
Nef	B8101	L	L76T/V	E	R	0.000		0.018			
Nef	B5801	A	A83G	E		0.159		0.003	11.33	26.74	0.0008
Nef	B4403	Y	Y102H	F		0.000		0.000			
Nef	B08	K	K105	E				0.178	−23.73	19.71	0.0001
Nef	C0701	K	K105R	E	R	0.000	0.124	0.000	21.77	23.34	0.0290
Nef	B4403	E	E108D	E		0.000		0.011			
Nef	B18	E	E108D	E		0.000		0.134			
Nef	C0602	Q	Q125H	E	R	0.149		0.030			
Nef	B42	P	P129	E				0.150	−15.22	22.70	0.0036
Nef	B53	V	V133I	F				0.078	6.21	21.38	0.0240
Nef	A23	F	F143Y	F		0.000		0.000			
Nef	C06	S	S187		R			0.104			

The table shows the results of the association analysis between HLA Class I alleles and HIV viral polymorphisms in HIV Nef. The columns of the table detail the HIV protein, the associated HLA Class I allele and viral polymorphism, whether the association lies in, or within, the flanking region of a known restricted epitope and whether this association is expected to revert in HLA-mismatched hosts. The q values for the statistically significant associations for the whole cohort, the ‘high’ CD4 count subgroup (>500 cells/µl) and ‘low’ CD4 count subgroup (<100 cells/µl) are shown, followed by the log_2_-adjusted odds ratios for the ‘high’ and ‘low’ groups. In the final column, the p value is reported, where there is a significant difference between the two odds ratios.

**Figure 2:**
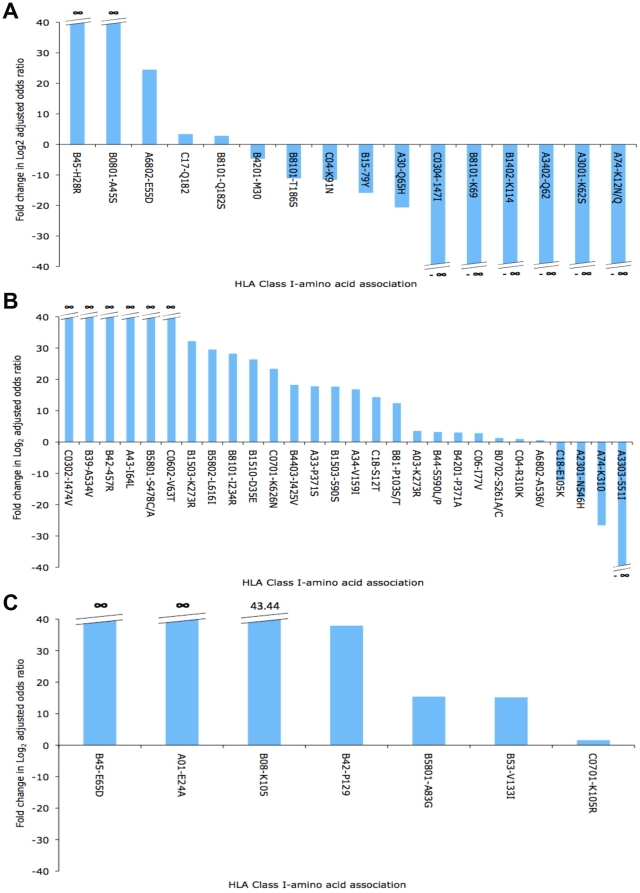
Changes in associations between HLA Class I alleles and HIV amino acid polymorphisms for patients with CD4 cell counts <100 cells/µl compared with patients with CD4 cell counts >500 cells/µl. The change in the strength of associations between HLA Class I alleles and specific HIV amino acid polymorphisms in patients with high and low CD4 cell counts is shown for HIV-1 (a.) Gag, (b.) Pol and (c.) Nef. Change in strength of the association is reported on the Y-axis as a log(2)-adjusted value. Associations are recorded on the X-axis according to the restricting HLA Class I allele and the specific viral polymorphism – e.g. B45-H28R in Gag means that in the presence of HLA B*45, Gag amino acid 28 mutates from a Histidine (H) to an Arginine (R). In this case there is at least a 40-fold change in the log(2)-adjusted odds ratio for this association in patients with CD4 counts <100 cells/µl compared with less progressed patients with CD4 cell counts >500 cells/µl. (All fold changes are capped at a value of 40).

We identified 40, 91 and 20 associations, respectively, between an HLA Class I allele and a viral polymorphism in the HIV-1 *gag*, *pol* and *nef* genes, in either, or both of, the ‘high’ or ‘low’ CD4 count subgroups. Of these, 16, 28 and 7 associations in HIV-1 *gag*, *pol* and *nef*, respectively, had significantly different log_2_-adjusted odds ratios in the two subgroups. These 51 associations are shown in Figure 2, according to HIV-1 gene. In *gag*, *pol* and *nef*, respectively, there were 5/16 (31%), 24/28 (86%) and 7/7 (100%) associations that were more prevalent in the ‘low CD4’ group. These data show that, where we could detect a difference, associations were more prevalent at lower CD4 counts in *pol* and *nef*, suggestive of continued selection pressure, but that certain associations – particularly in HIV-1 *gag* – were weaker. Why might HLA associations become weaker as disease progresses? One possible explanation is that as CTL pressure weakens due to immune exhaustion and loss of breadth, escape mutations with replicative fitness costs are no longer required by the virus. These therefore revert back to wild-type, resulting in a reduction in the observed log_2_-adjusted odds ratio. Thus, low-cost mutations accumulate over the course of infection, leading to higher prevalence in Nef and Pol, whereas high-cost mutations begin reverting, leading to a lower prevalence in Gag.

If changes in selection pressure were resulting in an increase or decrease in the prevalence of wild type virus during late-stage disease, we would expect to find changes in viral replicative capacity in patients with low CD4 cell counts. We therefore undertook assays of viral fitness. As the HIV *gag* gene was associated with the most potential examples of immune relaxation we implemented a fitness assay targeting the *gag* gene and focussed on three beneficial HLA Class I alleles (HLA B*57, HLA B*5801, and HLA B*8101) and one allele associated with more rapid progression, HLA B*5802. In particular, we highlighted two associations: the T186S mutation in the TPQDLNTML (TL9) epitope, restricted by B*8101, with an observed decrease in log_2_-adjusted odds ratio from 18.38 to 7.25 in patients with high and low CD4 cell counts, respectively, (consistent with immune relaxation, p = 0.0397, likelihood ratio test), and the B*57/B*5801 restricted epitope TSTLQEQIAW (TW10), with log_2_-adjusted odds ratios of 25.4 and 23.2 for high and low CD4 cell count stratification analysis (p = 0.12, likelihood ratio test, showing no significant evidence of immune relaxation). We focussed on these two associations as published data suggest that they are both associated with significant viral fitness costs [32] [18], [27] and yet in this dataset they behave very differently in terms of changes in log_2_-adjusted odds ratios.

### Increased viral replication capacity (VRC) of chimeric HIV-1 NL4-3 at low CD4 counts and high plasma viral loads

To investigate further possible reasons for declining selection pressure within Gag epitopes with progression to AIDS, we considered the hypothesis that loss of CD8+ T-cell responses as CD4 count declines results in reversion of escape mutants, with resulting increase in viral replicative capacity. To investigate the impact of the *gag-protease* mutations selected by different HLA class I alleles at different clinical stages, we constructed 148 chimeric HIV-1 NL4-3 viruses containing autologous *gag-protease* from patients of the Bloemfontein cohort. Patients with specific HLA class I alleles previously associated with control of HIV in this population (HLA-B*57/5801/8101) or lack of control (B*5802) [6] were selected for chimeric virus construction and stratified by CD4 cell count. 88 viruses were constructed from patients with low CD4 cell counts (<100 cells/µl), comprising HLA-B*57 (n = 2), HLA-B*5801 (n = 15), HLA-B*5802 (n = 54) and HLA-B*8101 (n = 10) plus 14 ‘neutral/other’ (i.e. neither protective or disadvantageous) HLA Class I alleles. From patients with high CD4 cell counts (>500 cells/µl), 42 viruses were made: HLA-B*57 (n = 7), HLA-B*5801 (n = 8), HLA-B*5802 (n = 15), HLA-B*8101 (n = 10) plus 10 ‘neutral/other’ HLA Class I alleles. Sixteen patients co-expressed two of these HLA class I alleles. A further 18 viruses were made from patients randomly selected from intermediate CD4 T cell counts. The VRC of the chimeric virus was expressed as percentage growth rate compared to the pNL4-3Δ*gag-protease* plasmid recombined with the wild type HIV-1 NL4-3 *gag-protease* insert.

**Figure 3:**
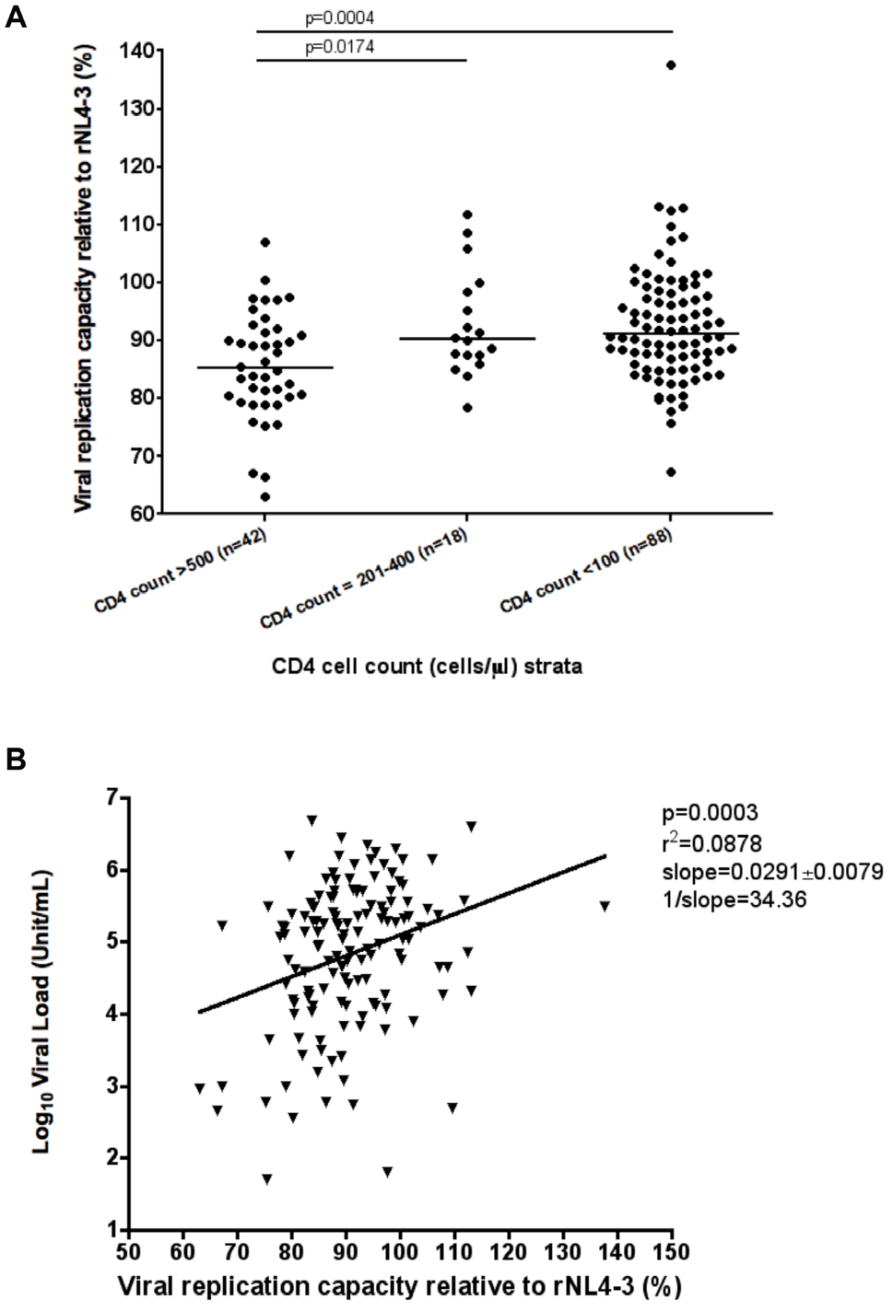
Viral replication capacity (VRC) according to CD4 cell count and plasma viral load. The viral replicative capacity (VRC) of chimeric NL4-3 viruses recombined with patient autologous gag-protease genes is compared with a laboratory recombinant HIV-1 NL4-3 strain. In (a.), The VRC of isolates from the Bloemfontein cohort is presented stratified by CD4 T cell count. The p-values are calculated using the Mann-Whitney test. In (b.), plasma viral load (log_10_ copies/ml) is correlated against the VRC from samples from the Bloemfontein cohort. In both panels VRC is presented as a percentage relative to the fitness of the control NL4-3 virus.

Initially we determined whether CD4 cell count alone was associated with VRC. Figure 3a shows that the VRC of the chimeric viruses from patients from different CD4 T cell count strata was significantly different (Figure 3a). The VRC from viral isolates from patients with the lowest CD4 T cell counts (<100 cells/µl, median 91.15%; IQR: 86.1–98.0%) was significantly higher than from patients with high CD4 T cell counts (>500 cells/µl, median 85.19%; IQR: 79.8%–91.8%), p = 0.0004 (Mann-Whitney test), showing that progression to AIDS is associated with an increase in viral fitness.

There was also a weak but statistically significant positive correlation between VRC and log_10_ plasma viral load (Figure 3b, p = 0.0003, r^2^ = 0.088, n = 142). The slope of the best-fit values (0.029±0.0079) indicated that a 34.4% increase in VRC accounted for an increase of one log_10_ in plasma viral load.

### VRC of chimeric *gag-protease* NL4-3 viruses varies according to HLA class I

**Figure 4:**
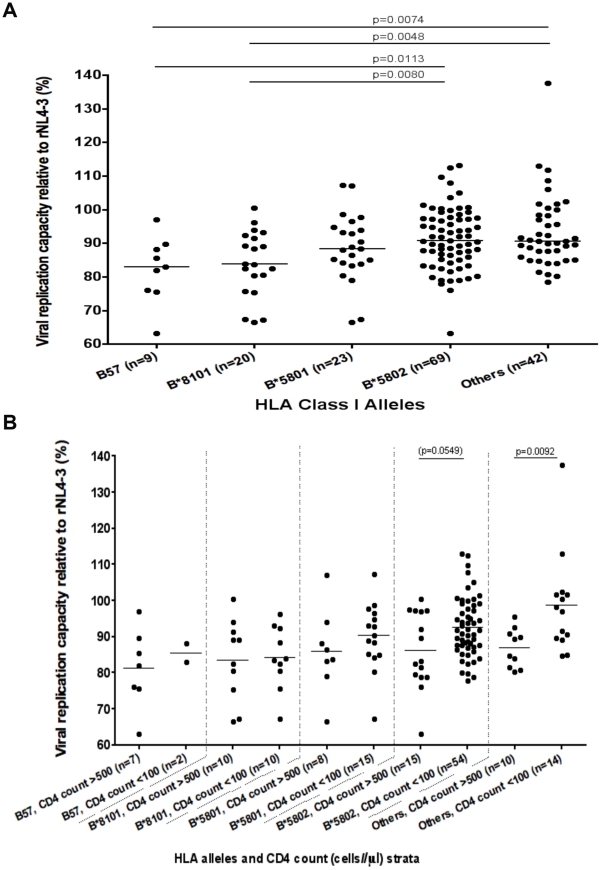
Viral replication capacity according to HLA Class I and CD4 cell count. (a) Viral replication capacity (VRC) of chimeric NL4-3 viruses containing patient autologous gag-protease stratified by HLA Class I. The VRC is expressed as percentage growth rate compared to control strains. The p-value is calculated using Mann-Whitney test. Panel (b.) shows further stratification according to CD4 cell count. The y-axis depicts VRC, expressed as percentage growth and the x-axis depicts HLA class I, with each HLA class I category divided further into “low” CD4 T cell count strata (<100 cells/mm3) and “high” CD4 T cell count (>500 cells/mm3). The p-value is calculated using Mann-Whitney test.

As higher VRCs were associated with higher plasma VL and lower CD4 cell count, we examined how this effect varied according to HLA Class I (Figure 4a). The VRC of chimeric viruses derived from patients with the protective alleles HLA B*57 (median = 83%, IQR: 76–89%; n = 9) and HLA B*8101 (median = 84%, IQR: 78–92%; n = 20) was significantly lower than from patients with ‘neutral/other’ HLA class I alleles (median = 91%, IQR: 81–92%; n = 26); (p = 0.007 and p = 0.005, respectively), or from patients with HLA B*5802, associated with more rapid clinical progression (median = 91%, IQR: 85–98%, n = 68) (p = 0.010 and p = 0.008, respectively, Mann-Whitney test).

To determine whether the association between HLA Class I and viral fitness was additionally impacted by disease stage we stratified the VRCs according to high and low CD4 cell counts (Figure 4b). This sub-group analysis shows that the mean VRC values are higher for each HLA Class I allele in the low CD4 count group suggestive of an increase in viral fitness in advanced disease, although in most cases these values are not statistically significant. This is likely to be an effect of being underpowered, as patient numbers become smaller with each sub-categorisation. Interestingly, there is a significant difference in the VRCs of viruses in the non-protective ‘neutral/other’ HLA Class I alleles (p = 0.0092, Mann-Whitney test), showing that although there is an HLA-specific effect on viral fitness, there is an additional association with CD4 cell count, independent of the more beneficial HLA Class I alleles.

### The impact of specific mutations on VRC in the context of HLA-B*8101 and HLA-B*B57/B*58

These analyses have shown that viral fitness is greater in viruses from patients with advanced disease, and that certain protective HLA Class I alleles (such as B*57, B*8101 and B*5801) are associated with greater fitness costs. The HLA-association analysis presented above (Tables 1,2 and 3) revealed that in patients with low CD4 counts HLA B*57/*5801 was associated with persistence of T242N in the TW10 epitope, whereas HLA B*8101 was associated with a potential loss of selection pressure reflected by the decreased prevalence of T186S mutants in the TL9 epitope. We therefore focused further on these well-characterised escape mutants to investigate how they impacted viral fitness at different stages of disease progression.

**Figure 5:**
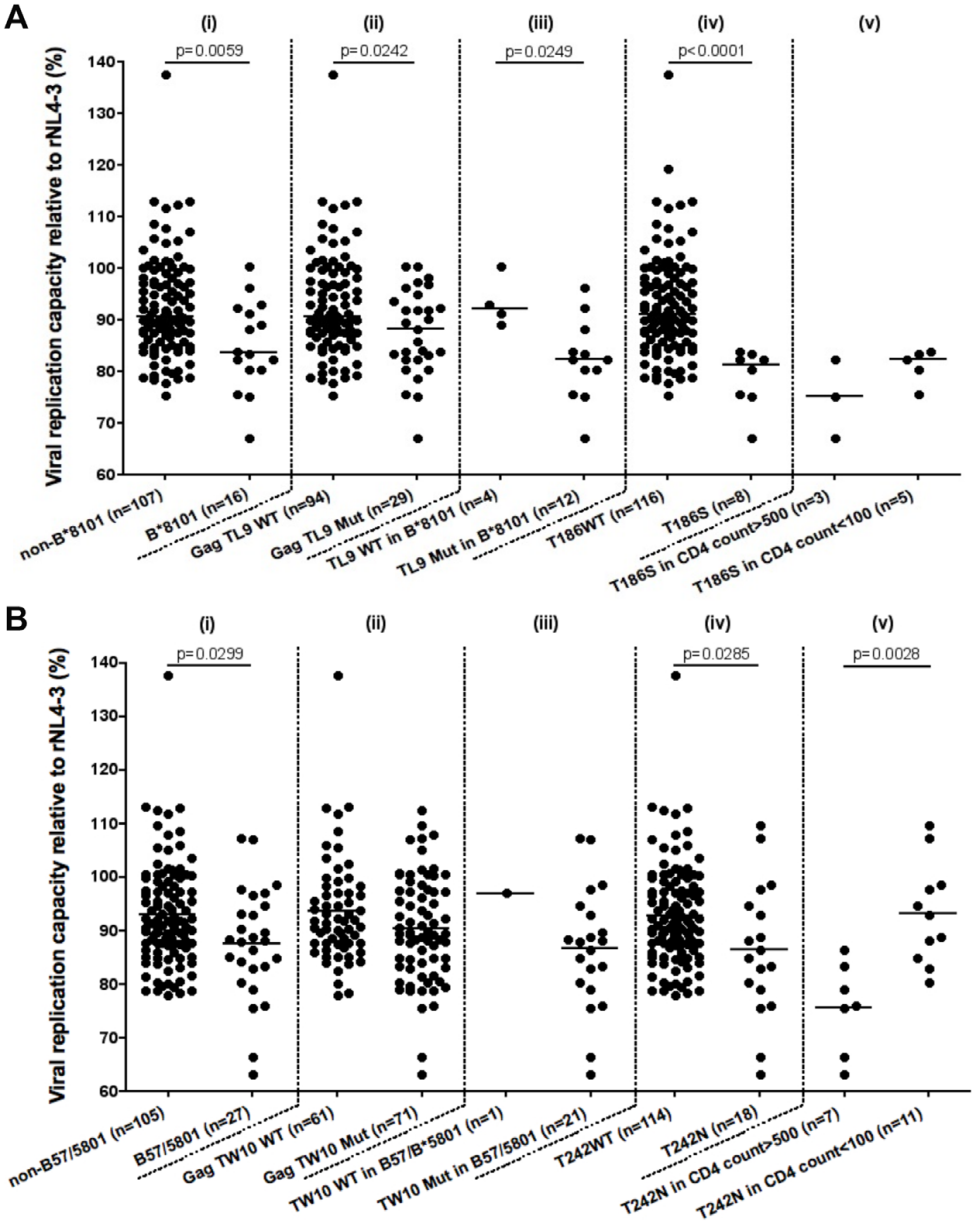
Viral replication capacity (VRC) in viruses with mutants in (a) Gag TL9 and (b) Gag TW10 according to HLA Class I and CD4 cell count. In panel (a), the y-axis depicts viral replication fitness (VRC) of chimeric NL4-3 viruses containing patient autologous *gag-protease*, expressed as percentage growth rate compared to a control recombinant NL4-3 strain. The x-axis depicts the stratification of patients according to (i) HLA-B*8101, (ii) polymorphic status within the *gag* TL9 epitope in all patients, (iii) polymorphic status of *gag* TL9 in patients with HLA B*8101, (iv) presence of T186S in *gag* in Gag TL9, and (v) T186S further stratified according to the CD4 T cell count (<100 cells/µl or >500 cells/µl). The p-value is calculated using Mann-Whitney test. In (b), patients are stratified according to (i) HLA-B*57 or B*5801 alleles, (ii) polymorphic status within the *gag* TW10 epitope, (iii) polymorphic status of TW10 in patients with HLA-B*57 or B*5801 alleles, (iv) polymorphic status of amino acid position 242 (within TW10 epitope – T242N) in all patients and (v) by T242N in patients with HLA-B*57 or B*5801 according to CD4 count. The viral isolates possessing other known costly escape mutations (A163G and T186S) are excluded from this analysis. The p-value is calculated using Mann Whitney test.

For HLA-B*8101, we excluded from the analysis any viruses with key mutations restricted by other HLA Class I alleles (that is, T242N and A163G) which were known to impact fitness. Viruses from patients with HLA B*8101 were significantly less fit than viruses from patients in the rest of the cohort (p = 0.0059, Mann-Whitney test; Figure 5a) and mutations in the B*8101-restricted Gag TL9 epitope were associated with lower VRCs (p = 0.024) both in the whole cohort and in patients with HLA B*8101 (p = 0.025). The main single mutation conferring loss of replicative fitness was the T186S immune escape mutation (p<0.0001). Neither the Q182S or T190X mutations had an impact on VRC in this cohort (data not shown). Although patient numbers were small, there was no significant difference between the VRCs of viruses with the T186S mutation according to high and low CD4 count stratification. These data show that although B*8101 imposes a fitness cost on the virus through selection of T186S, we found no evidence that, when this mutation persists, the fitness cost is restored at low CD4 cell counts.

We compared the fitness costs associated with HLA B*8101 with those associated with B*57 and B*5801, as the HLA-association analysis suggested that for these latter alleles escape mutations persisted in advanced disease. We pooled the data for patients with HLA B*57 and B*5801 as these alleles have highly related binding motifs, and both present the Gag TW10 epitope and select the T242N escape mutation [36]. As for HLA B*8101, viruses from patients with HLA-B*57 or HLA-B*5801 were significantly less fit than those from the rest of the cohort (p = 0.0299, Mann-Whitney test; Figure 5b), supporting the association between protective HLA Class I alleles and viral replicative cost (Figure 4a). In the whole cohort there was no significant effect of all mutations in the TW10 epitope on VRC. For patients with HLA B*57 or B*5801, there was a non-significant trend for all mutations within the TW10 epitope to impair fitness, although the power of this analysis was limited as only one of the 22 patients had not selected a mutant epitope. However, the fitness cost of the T242N mutation was confirmed when the whole cohort was analysed, when it was associated with a significant drop in VRC (p = 0.0285). This result was independent of the effect of mutations in other B*57/B*5801 associated epitopes such as Gag KF11 or Gag IW9 (p = 0.024; data not shown), showing that the fitness cost was associated with T242N, rather than reflecting being positive for HLA B*57/B*5801.

In the HLA-association analysis we had found that T242N was maintained in late disease, even though these data, as well as previous reports, suggest that this mutation is associated with a fitness cost. We explored this apparent discrepancy by stratifying the fitness analysis of T242N according to CD4 count (Figure 5b), and found that in patients with advanced disease (CD4 counts <100 cells/µl), viral fitness had been restored even though the T242N persisted (p = 0.0028).

### Compensatory mutations accrue in advanced HIV infection in patients with HLA B*57 and B*5801

**Figure 6:**
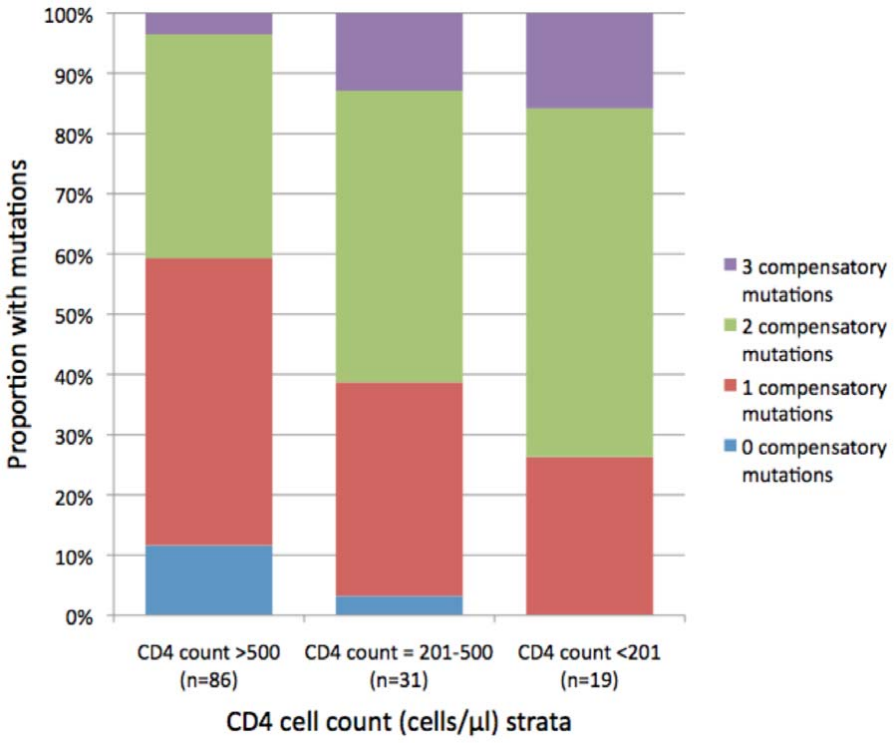
Prevalence of compensatory mutations with the T242N mutation in the HLA-B57 and B*5801 restricted epitope, Gag TW10. In the 100% stacked column, the y-axis shows the number of mutations accumulated at the three Gag protein amino acid sites (H219Q, I223V and M228I) that compensate for the T242N mutation [27]. The x-axis depicts the categorical CD4 T cell count strata and the number of patients within each strata (the “High CD4” group refers to patients with CD4 T cell counts >500 cells/µl, the “Intermediate CD4” group refers to patients with CD4 T cell counts between 200 and 400 cells/µl, and the “Low CD4” group refers to patients with CD4 T cell counts <100 cells/µl).

As the T242N mutation has been previously linked with compensatory mutations we analysed our cohort to see if these accrued in late stage disease to explain the rise in viral fitness despite the maintenance of T242N. For the T242N mutation in TW10, compensatory mutations have previously been published (H219Q, I223V and M228) [27], and so we examined the frequency of these mutations in the different CD4 T cell count strata (Figure 6). We found a cumulative increase in the number of compensatory mutations in patients with T242N in individuals with lower CD4 cell counts, consistent with the increase in fitness in the previous analyses (P = 0.013).

Together these data suggest that advanced HIV disease is associated with an increase in viral fitness, which may contribute to the rise in plasma viral load frequently seen in AIDS. However, this increase in fitness is multifactorial and we report two processes specific to different HLA Class I alleles – reversion due to immune relaxation and compensatory mutations – to potentially explain these findings.

## Discussion

In this study, we have explored the hypothesis that progression to AIDS is associated with a rise in viral replicative capacity, and the mechanisms associated with this. We proposed that a rise in viral fitness in AIDS could be due either to a relaxation of CTL-imposed selection pressure resulting in the reversion of costly mutations or, alternatively, if immune pressure is maintained, compensatory mutations might restore the fitness costs of persisting escape mutations.

We initially tested the hypothesis by looking for evidence of changes in T cell-imposed selection pressure measured by gamma interferon ELISPOT assays. We found that the breadth of the response narrowed as the CD4 count declined and that, specifically, the contribution of Gag responses to the overall response decreased. It has previously been reported that acute HIV infection is associated with narrow, high affinity T cell responses which broaden as patients progress into chronic infection [37]. Here, we see the reverse of this process with a return to a much narrower range of T cell responses. It would also be interesting to measure whether there was a loss of high affinity T cells in the advanced stage patients in this cohort as this might be compatible with a weakening in selection pressure.

Although we identified a narrowing of the T cell responses in AIDS, the ELISPOT data alone are not sufficient to infer changes in selection pressure. We therefore looked for evidence of differential selection in patients with high and low CD4 cell counts using a statistical HLA Class I association study. By combining sequence and HLA data from the Bloemfontein cohort and the Durban cohort, we were able to define two groups of patients with CD4 counts less than 100 cells/µl (n = 196) and greater than 500 cells/µl (n = 299). These CD4 count strata were defined to represent extremes of HIV-related disease without reducing the power of the analysis. The WHO have defined that antiretroviral therapy should be commenced at a CD4 count of 350 cells/µl. Below 200 cells/µl, opportunistic infections become more common. A count of 100 cells/µl or below, therefore, represents advanced HIV disease and progression to AIDS. Above 500 CD4 cells/µl it would be highly unusual for there to be opportunistic infections and the most recent NIH guidelines do not recommend treating above this level [38].

To identify mutations in the HIV genome associated with individual HLA Class I alleles we used an established technique using phylogenetic dependency networks [31], which has been widely implemented in the HIV literature [12], [14], [35], [39]. In this approach, the risk of error due to viral founder effects and misidentification of co-variant sites is corrected for by integrating the phylogeny of the sequences into the analysis. Identified associations are hypothesised to be the result of cytotoxic T cell imposed selection pressure and in other analyses such associations have been proven experimentally. Although there have been a number of HLA-association studies reported, none have stratified patients according to disease progression and compared the strength of selection in different strata according to log_2_-adjusted odds ratios.

In our cohorts we identified 151 HLA-associated mutations in HIV-1 *gag*, *pol* or *nef* that were significant in patients with either high or low CD4 cell counts. By comparing log_2_ adjusted odds ratios in the CD4 strata, we found that there was evidence for both immune relaxation and intensification in patients with low CD4 counts compared with high CD4 counts. This suggests that for some HLA Class I–restricted responses the selection pressure imposed by CTL is maintained at very low CD4 cell counts but that for others there may be reversion of escape mutations associated with a reduction in selection pressure. Interestingly, the majority of events compatible with immune relaxation were in HIV Gag, which was also the protein for which there was a relative narrowing of T cell ELISPOT responses in AIDS patients.

Two key sites potentially reflecting immune ‘persistence’ and ‘relaxation’ were the HLA B*57/B*5801 associated T242N mutation and the B*8101 associated T186S mutation, respectively. For the former, the association analysis provided evidence for on-going mutation associated with compensatory mutations, whereas for the latter there was evidence for reversion. A limitation of this study is that it is cross-sectional rather than longitudinal, potentially biasing our data interpretation. An alternative explanation for immune relaxation is that the patients who progress are the ones who do not make an immune response and so do not select mutants, therefore enriching patients with low CD4 counts with wild-type viruses. This would be compatible with the finding that targeting certain key epitopes may be associated with different speeds of clinical progression [40], but does not explain the eventual outcome of those patients enriched for immune escape mutations with high CD4 cell counts. To determine which occurs would require a prospective longitudinal study of untreated progression to AIDS. In our cohort, analyses of HLA B*8101 positive individuals with data on ELISPOT responses and viral sequence, show that for patients with CD4 counts between 200–500 cells/µl, 50% (3/6) who did not recognise TL9 carried the T186S mutant. In contrast, for patients with CD4 counts <100 cells/µl there were no ELISPOT negative patients with T186S (0/2), suggesting that these had reverted rather than having been consistently wild type, although in this study numbers are too small to be conclusive.

Viral fitness was measured using a recombinant assay in which a patient-derived RT-PCR amplicon of HIV-1 *gag-protease* was inserted into a *gag-protease*-deleted pNL4-3 plasmid. The advantage of this approach is that it uses the full *gag* gene from the patient sequence as well as the associated *protease* that encodes the enzyme for cleavage of the Gag-Pol protein. The disadvantage of this assay is that it is in a parallel rather than competitive format, and therefore may lack sensitivity for small fitness differences. In addition, the construct is a recombinant of a subtype B backbone with a subtype C insert. Although this approach has been published elsewhere [32], any data should be interpreted relative to the control virus rather than as an absolute measure of viral fitness. Choice of control is important. We used a subtype B wild-type backbone (pNL4-3Δ*gag-protease*) into which we recombined the pNL4-3 *gag-protease*. This controlled for any change in fitness due to the recombination step.

We found that VRC was significantly greater in patients with low CD4 cell counts and there was a significant, although weak, correlation with plasma viral load. These data, which are consistent with a population-based study of HIV subtype B-infected individuals [33], are amongst the first supporting a link between viral fitness and plasma viral load, and show that this *ex vivo* assay reflects viral replication *in vivo*. Analysing our data by HLA Class I revealed that HLA Class I alleles that are associated with clinical advantage (B*8101, B*57, B*5801) were associated with impaired viral fitness, as had been inferred from previous analyses of the reversion of transmitted escape mutations in HLA mismatched hosts [22], [23]. Interestingly, however, the increase in viral fitness with lower CD4 cell counts did not appear to be restricted to beneficial HLA alleles and was also present in a selection of viruses from patients with ‘neutral/other’ alleles.

The HLA-amino acid association analysis showed that for HLA B*8101 there was a decrease in mutations in the Gag TL9 epitope at low CD4 cell counts, whereas for HLA B*57/B*5801 there was persistence of the T242N escape mutation in TW10. The fitness assays showed that the T186S mutation in TL9 was associated with a fitness cost (P<0.0001) and it is therefore possible that in patients with B*8101 and low CD4 counts, the decrease in prevalence of the T186S mutation reflects reversion to a fitter strain coincident with the weakening of T cell responses. For B*57/B*5801 we saw a different pattern. Here, possession of HLA B*57/B*5801 was associated with less fit viruses in conjunction with the T242N mutation in the Gag TW10 epitope, but in patients with low CD4 counts fitness was restored even though T242N persisted. We therefore sought compensatory mutations to explain this finding. For T242N there are four documented compensatory mutations at Gag codons 219, 223, 228 and 248 [27]. For this analysis of subtype C HIV-1 we excluded codon 248, as the 248A variant represents the subtype C consensus. When we stratified patients with T242N according to CD4 strata we found a significant (p = 0.013) increase in numbers of compensatory mutations at lower CD4 counts, consistent with the rise in VRC.

These data show that viral fitness increases with progression to AIDS. Whether this is cause or effect is difficult to determine, although the fact that we see evidence for both reversion and compensatory mutations in the same cohort indicates that progression may be a mixture of the two. What is clear is that viral fitness is a significant component of progression to AIDS and, as such, should be considered as a target for intervention, for example through vaccines aimed at epitopes which escape with high fitness costs. Trials such as DART have shown that antiretroviral therapy can be extremely effective in regions such as sub-Saharan Africa [41], however the costs and logistics of provision are complex. Any intervention that could prevent new infections or delay a requirement for therapy could have major economic and health implications and therefore combining such a vaccine with antiretroviral provision could be a productive strategy.
